# Impact of Left Ventricular Assist Devices on Days Alive and Out of Hospital in Hemodynamically Stable Patients with End-Stage Heart Failure: A Propensity Score Matched Study

**DOI:** 10.3390/life12121966

**Published:** 2022-11-24

**Authors:** Theresa Tenge, Sebastian Roth, René M‘Pembele, Giovanna Lurati Buse, Florian Boenner, Christina Ballázs, Igor Tudorache, Udo Boeken, Artur Lichtenberg, Martin Neukirchen, Ragnar Huhn, Hug Aubin

**Affiliations:** 1 Department of Anesthesiology, Medical Faculty and University Hospital Duesseldorf, Heinrich-Heine-University Duesseldorf, 40225 Düsseldorf, Germany; 2 Department of Cardiology, Pulmonology and Vascular Medicine, Medical Faculty and University Hospital Duesseldorf, Heinrich-Heine-University Duesseldorf, 40225 Düsseldorf, Germany; 3 Department of Cardiac Surgery, Medical Faculty and University Hospital Duesseldorf, Heinrich-Heine-University Duesseldorf, 40225 Düsseldorf, Germany; 4 Department of Anesthesiology, Kerckhoff Heart and Lung Center, 61231 Bad Nauheim, Germany

**Keywords:** heart failure, cardiac surgery, heart transplantation, left ventricular assist devices, patient-centered outcomes

## Abstract

The two main surgical options to treat end-stage heart failure are heart transplantation (HTx) or left ventricular assist device (LVAD) implantation. In hemodynamically stable patients, the decision for HTx listing with or without LVADs is challenging. We analyzed the impact of both options on days alive and out of hospital (DAOH) and survival. This retrospective study screened all patients with HTx or LVAD implantation between 2010 and 2020. The main inclusion criterion was hemodynamic stability defined as independence of intravenous inotropic/vasoactive support at decision. Propensity score matching (PSM) was performed. The primary endpoint was DAOH within one year after the decision. Secondary endpoints included survival, duration until HTx, and hospitalizations. In total, 187 patients received HTx and 227 patients underwent LVAD implantation. There were 21 bridge-to-transplant (BTT)-LVAD patients (implantation less than a month after HTx listing or listing after implantation) and 44 HTx-waiting patients included. PSM identified 17 matched pairs. Median DAOH at one year was not significantly different between the groups (BTT-LVAD: median 281, IQR 89; HTx waiting: median 329, IQR 74; *p* = 0.448). Secondary endpoints did not differ significantly. Our data suggest that BTT-LVAD implantation may not be favorable in terms of DAOH within one year for hemodynamically stable patients compared to waiting for HTx. Further investigations on quality of life and long-term outcomes are warranted.

## 1. Introduction

In end-stage heart failure, heart transplantation (HTx) remains the gold-standard therapy according to American and European guidelines [1,2]. However, left ventricular assist devices (LVADs) have not only shown improved survival and quality of life compared to optimized pharmacological treatment [3,4], but also show comparable survival to HTx [5,6]. From a patient point of view, the decision to solely wait for the gold-standard HTx or to undergo LVAD implantation with its possible risks and complications as a bridge to transplant (BTT) is absolutely crucial. Especially in hemodynamically stable patients, clinicians are often confronted with the decision of listing their patients for HTx with or without BTT-LVAD implantation [7].

Within the Eurotransplant region, patients in an acute life-threatening status can be “high-urgency (HU) listed” on the HTx waiting list, while stable patients being “t-listed” on the regular waiting list often have to wait for elective HTx for a much longer period. Patients receiving a LVAD implantation can be further classified using the Interagency Registry for Mechanically Assisted Circulatory Support (INTERMACS) profile. INTERMACS 1 to 3 typically indicate a patient who is dependent on vasoactive or inotropic therapies and represent the majority of patients receiving LVAD implantation [8]. Studies focusing on patients with an INTERMACS 4 who are not inotropic-dependent, only on oral medication and therefore stable in their end-stage heart failure, are rare. A re-evaluation of the ROADMAP study compared INTERMACS 4–7 patients to optimal medical management and favored LVAD therapy, especially in INTERMACS 4 regarding survival and health-related quality of life [9]. However, adverse events are still more frequent in LVAD compared to medical therapy only [6,7,9,10,11]. Despite this, it is important to note that the recent developments in the pumps with a centrifugal continuous flow significantly improved survival free of a debilitating stroke or reoperation [12,13]. A comparison of hemodynamically stable end-stage heart failure patients awaiting HTx on the regular waiting list and INTERMACS 4 patients receiving a BTT-LVAD has not yet been performed. In addition, data on the life impact and quality of life of LVAD implantation in these patients are scarce. Days alive out of hospital (DAOH) has been proposed as a more patient-centered outcome in this context, which is easy to measure, readily available, statistically efficient, and cost-effective [14,15].

The primary aim of this study was to compare DAOH in hemodynamically stable patients awaiting HTx on the regular waiting list vs. patients undergoing BTT-LVAD implantation. We hypothesized that BTT-LVAD patients have more DAOH than patients solely waiting for HTx. We also aimed to characterize the different trajectories of these patients after the decision.

## 2. Materials and Methods

### 2.1. Study Design

This study was a retrospective single-center study conducted at University Hospital Duesseldorf, Germany. Ethical approval was obtained for studying both, the LVAD and HTx databases, by the local ethics committee (reference numbers: 2020-1058 and 4567). Written informed consent for this analysis could be waived due to the retrospective nature of the study, but all patients gave written informed consent in advance to be registered in a dedicated prospective local database. This article was written to strengthen the reporting of the observational studies in epidemiology (STROBE) checklist for retrospective cohort studies.

### 2.2. Patients

**Figure 1:**
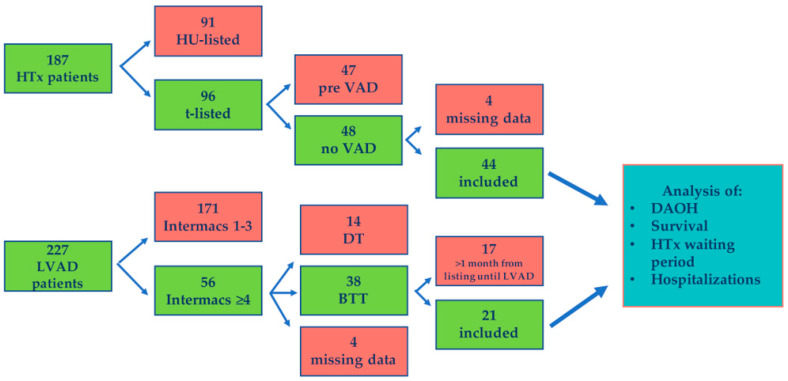
Study flowchart. Process of data search in the two databases, HTx and LVAD. Patients who followed up and were finally included are marked in green. Marked in red are the patients who were excluded in the respective databases. HTx = heart transplantation; HU = high-urgency status; (L)VAD = (Left) ventricular assist device; Intermacs = Interagency Registry for Mechanically Assisted Circulatory Support; DT = destination therapy; BTT = bridge to transplant; DAOH = days alive and out of hospital.

All patients aged ≥18 years who received LVAD implantation or HTx at University Hospital Duesseldorf between September 2010 and December 2020 were screened from the databases. Patients that were listed for a HTx but died before receiving a HTx were not included in the database. Hemodynamically stable BTT-LVAD patients (≥INTERMACS 4) and hemodynamically stable HTx patients on the regular waiting list theoretically eligible for ventricular assist device therapy at the time of listing were included. Hemodynamic stability was defined as the independence of intravenous inotropic or vasoactive support at the time of LVAD implantation or sole HTx listing, respectively. If the time difference between t-listing and LVAD implantation was more than one month, patients were excluded. HTx listing after BTT-LVAD implantation was no exclusion criterion. Thus, we aimed to avoid bias from patients who have had LVAD implantation due to a prolonged HTx waiting time. Patients with LVAD as destination therapy (DT) due to a common contraindication for HTx and patients with missing data or incomplete medical records regarding the endpoints of interest were also excluded. Data on patient characteristics, medical history, and hospitalizations within one year were extracted from electronic medical charts and entered in the local and continuously updated database. Information on the study selection process can be found in Figure 1.

### 2.3. Propensity Score Matching

As the groups were non-randomized, we performed a matched propensity score (PS) analysis. A logistic regression model including seven preoperative patient characteristics was used to estimate the PS. The PS matching was performed using a case–control matching algorithm with a caliper width of 0.2 standard deviations of the logit. Balance between patient characteristics before and after matching was assessed using 2-sample t-tests (continuous variables, after checking for normality) or chi-square tests (categorial variables). There is a lack of literature on decision criteria for HTx or BTT-LVAD in hemodynamically stable patients. Therefore, the following preoperative characteristics which might influence the decision for or against each treatment option were chosen to calculate the PS [15]: age, sex, body mass index (BMI), diagnosis (ischemic cardiomyopathy, dilatative cardiomyopathy, others), preoperative renal failure (defined as serum creatinine ≥1.5 mg/dL), New York Heart Association (NYHA) status, and the left ventricular ejection fraction (LVEF, measured via transthoracic echocardiogram). The LVEF was classified as preserved (>50%), mildly reduced (40–49%), and reduced (<40%).

### 2.4. Outcomes

The primary endpoint of this study was DAOH at one year after decision for HTx listing or after BTT-LVAD implantation. DAOH was calculated as previously reported [15] and measured as the sum of days in hospital for each patient subtracted from 365 days. If a patient died during the first year, the difference between days survived and 365 days was added to the sum of days in hospital before subtraction from 365. Hospitalizations were defined as planned or unplanned stays of at least one overnight stay in hospital. All end-stage heart failure patients were closely connected to our center; thus, we did not expect external hospitalizations without a note in the patient’s medical record. Secondary endpoints included the duration until HTx; reasons for delisting or HU listing; the amount, durations, and reasons for hospitalizations; as well as survival analysis.

### 2.5. Statistical Analysis

Statistical analysis was performed using IBM SPSS© software version 28.0 (IBM Corp., Armonk, NY, USA) and Microsoft Excel 2020 (version 16.42, Microsoft Corp., Redmond, WA, USA). As this was a retrospective and exploratory data analysis, a formal sample size calculation was not implemented. Categorical data are presented as counts (*n*) with corresponding percentages (%). Continuous variables are reported as mean ± standard deviation (SD) or as median with interquartile ranges (IQR). *p* < 0.05 was considered as statistically significant. Due to the small sample size of this study, the Shapiro–Wilks test was used to test for normality and for choosing the appropriate statistical method. Based on the results of the Shapiro–Wilks test, a non-parametric Mann–Whitney U-test was used to compare DAOH in both groups. Null-hypothesis significant testing was conducted using Bayesian analysis for the Mann–Whitney-U statistics (JASP Team (2020). JASP (Version 0.14.1, Amsterdam, The Netherlands) [Computer software]). To compare the secondary endpoints, descriptive statistics as well as Kaplan–Meier analysis for survival comparison were performed.

## 3. Results

### 3.1. Propensity Score Matching

Between September 2010 and December 2020, a total of 187 patients underwent HTx and 227 patients underwent LVAD implantation at our center, respectively. Based on the in- and exclusion criteria, 21 BTT-LVAD patients (HeartMate II™: 2; HeartMate 3™: 7; Medtronic HVAD™: 12) and 44 HTx-waiting patients were included. Propensity score matching resulted in 17 matched pairs. In the matched BTT-LVAD group, two patients had a HeartMate II™, five had a HeartMate 3™, and ten patients were implanted with a Medtronic HVAD™ device. Table 1 summarizes the preoperative patient characteristics before and after matching. In both groups, patients were mostly male (BTT-LVAD: *n* = 13 (76.5%), HTx-waiting: *n* = 12 (70.6%)), between 50 and 60 years old, and around 30–40% had pre-existing renal failure. In most cases, the diagnosis leading to end-stage heart failure was ischemic cardiomyopathy. The majority of patients reported heart failure symptoms (NYHA ≥ 3) and median LVEF was 15%.

### 3.2. Primary Endpoint

**Figure 2:**
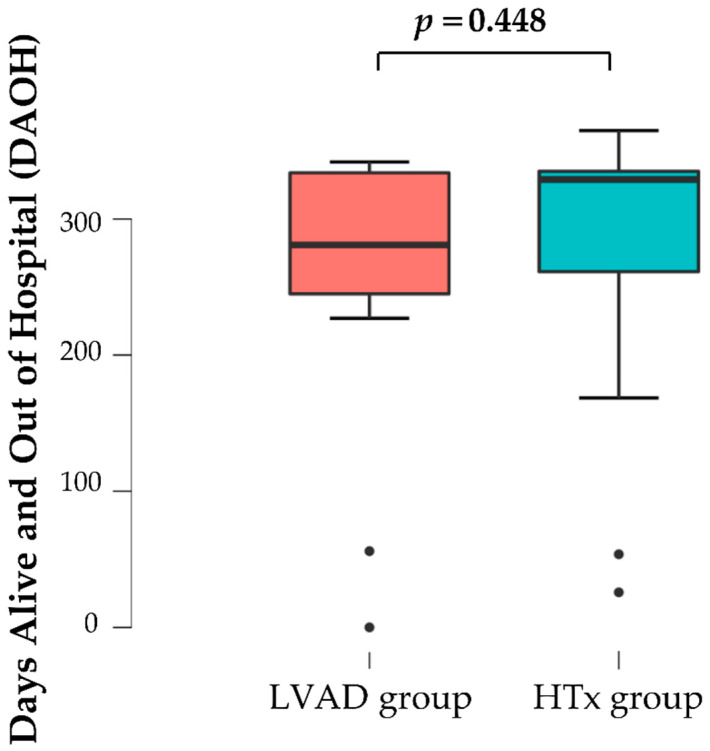
Days alive and out of hospital (DAOH) within one year in non-inotropic-dependent end-stage heart failure patients awaiting heart transplantation (HTx) on the regular waiting list and after bridge-to-transplant left ventricular assist device (LVAD) implantation.

Overall, median DAOH at one year was 313 (IQR 88) days, with no significant difference between the groups (BTT-LVAD group: median 281, IQR 89; HTx waiting group: median 329, IQR 74; *p* = 0.448, see Figure 2). The Bayes factor 1:2.246 indicates that an alternative hypothesis is 2.246 times less likely than a null hypothesis. In a sub analysis, we further compared all patients that did not receive a HTx in the first year after LVAD implantation or after t-listing on the waiting list. We excluded patients that were delisted in the first year. The DAOH was significantly higher in HTx-waiting patients compared to LVAD patients (BTT-LVAD group: *n* = 7, median 334, IQR 39; HTx waiting group: median 362, IQR 5; *p* = 0.025).

### 3.3. Secondary Endpoints

**Figure 3:**
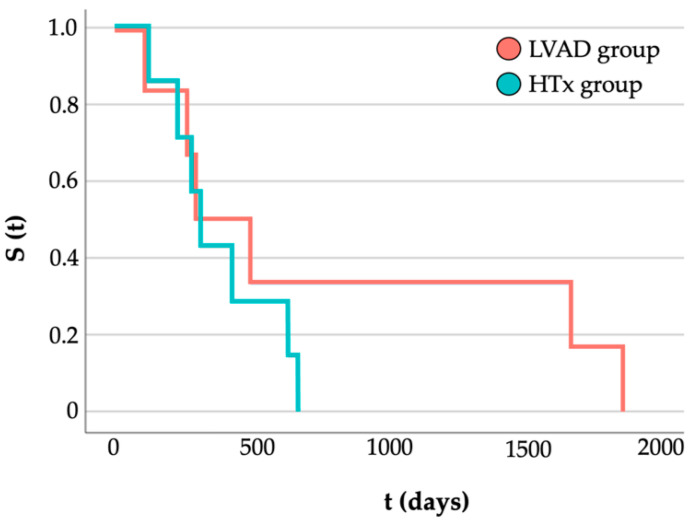
Survival rate (S) over time (t) of the deceased after left ventricular assist device (LVAD, *n* = 6, red) implantation or listing for heart transplantation (HTx, *n* = 7, green) on the regular waiting list in days as estimated according to the Kaplan–Meier method (log-rank test: *p* = 0.673).

In the first year after the decision for BTT-LVAD implantation or to wait on the regular waiting list, three LVAD patients and four patients on the HTx waiting list died. Survival analysis by the Kaplan–Meier method showed no significant difference between the groups at one year (BTT-LVAD group = 82% vs. HTx-waiting group = 76%; log rank: *p* = 0.673; Table 2). Figure 3 shows the survival rate of all deceased patients.

The median waiting time for HTx for patients without LVAD on the list was 179 (IQR 308) days with two patients being HU-listed in the process. In the first year after listing, 12/17 (70.6%) patients received a HTx. In the BTT-LVAD population, nine patients (52.9%) received a HTx after waiting for a median time of 256 (IQR 275) days after implantation (no statistical significance between the groups from t-decision until HTx: *p* = 0.651). In the first year, five patients (29.4%) were transplanted. Five patients were delisted and changed from BTT-LVAD to a DT concept due to increased incompliance and multimorbidity, and one patient due to intracerebral bleeding. Of the 17 included LVAD patients, 4 became HU-listed due to LVAD pump thrombosis, cerebral ischemia, or driveline infection. Because we needed to include not only matched patients in the analysis of LVAD-associated complications leading to a HU status, we also examined all 21 BTT-LVAD patients (unmatched). The additional four patients did not have any further HU listing due to LVAD complications.

Analyzing the amounts, durations and reasons of hospitalization within the first year revealed the following: the mean number of hospital stays in initially hemodynamically stable patients awaiting HTx in the first year after listing on the regular waiting list was 2.53 (SD 2.12) with a mean duration of 25.77 (SD 25.17) days. Other than the HTx in 12 patients, right or left heart catheterization, cardiac decompensations before HTx, and myocardial biopsies after HTx also led to the hospitalization of patients on the waiting list. In the first year after LVAD implantation, the patients’ mean number of hospital admissions was 3 (SD 1.97) with a duration of 32.24 (SD 28.39) days. Besides the five patients (29.4%) that received HTx in the first year and were therefore admitted longer, analyzing the LVAD patients revealed the following interesting result: five (29.4%) patients were only admitted for LVAD implantation and had no further hospitalizations in the following year. One patient died during the index LVAD-implantation admission due to right heart failure.

## 4. Discussion

This is the first study to compare the life impact between hemodynamically stable patients with end-stage heart failure that were either eligible for sole HTx listing or BTT-LVAD implantation. Supporting the decision-making process between both options through valid data is of great and immediate relevance for clinical practice. Therefore, we aim to help patients to evaluate and compare both options with relevant endpoints from their point of view. We used the available retrospective data to assess and compare DAOH at one year in both groups. Additionally, we analyzed survival, the HTx waiting period, and hospitalizations. Our findings show that BTT-LVAD implantation (maximum one month after HTx listing) as an end-stage heart failure patient who is still hemodynamically stable does not lead to a significant difference over sole HTx listing in terms of hospitalized days in the year after the decision and 1-year mortality. Nevertheless, further investigation on quality-of-life parameters and with larger cohorts should follow.

The systematic reviews and meta-analyses by Theochari and colleagues and Zhang and colleagues synthesized the published evidence on mortality and adverse events in HTx and BTT-LVAD patients and did not find any statistically significant difference (Theochari et al.—seven studies, no difference in 1-year mortality, pooled odd’s ratio (OR): 0.91, 95% confidence interval (CI): 0.62–1.32; Zhang et al.—twelve studies in total, two studies on five-year mortality with no significant differences, pooled OR: 1.02, 95% CI: 0.93–1.11, and no difference in stroke, bleeding, and infection adverse events) [5,16]. Additionally, ten-year survival does not show statistical significance in a recently published study (9420 patients with LVAD; 23,877 with direct HTx; 76% overall survival at ten years; *p* = 0.380) [17]. However, one included article in both reviews found the in-hospital mortality to be significantly higher for HTx waiting list patients compared to BTT-LVAD patients (42.3% versus 4.3%, *p* = 0.002) [18]. This last-mentioned study published by Attisani and colleagues compared patients on the waiting list for HTx with urgent conditions and LVAD patients with an INTERMACS 1 or 2 status and is therefore not comparable to our present study [18]. A study that included INTERMACS 4 patients and compared these to optimal medical treatment showed improved 1-year survival and health-related quality of life in the device group [9]. In our data, survival also did not differ between the HTx-waiting and BTT-LVAD patients. However, further studies with larger sample sizes and longer periods need to validate the effect on survival.

The life impact parameter DAOH itself was investigated in both HTx and LVAD patients separately [15,19]. In LVAD patients, the INTERMACS performance status and other parameters such as chronic kidney failure significantly influence DAOH [15]. The authors agreed to use these factors to optimize patient selection in LVAD treatment, therefore, we used these for PSM [15]. Our study did not find any statistical significance in DAOH at one year between the groups of stable HTx-waiting patients and INTERMACS ≥4 BTT-LVAD patients. One could draw the conclusion that HTx waiting might be superior to avoid LVAD-associated burdens and complications. However, LVAD implantation improves heart failure symptoms and the patient’s overall condition with increased blood flow of organs. We could see in our study that no hospitalizations occurred due to cardiac decompensations in the LVAD group. However, patients might suffer from LVAD-related complications and the waiting period for HTx is longer (not statistically significant). As the Bayes factor of the analysis is BF01: 2.246, the evidence for the null hypothesis is only anecdotal. Therefore, it is important to interpret the results of this study as a first approach to analyze therapeutic options for hemodynamically stable end-stage heart failure patients. Prospective studies to evaluate the impact of each invasive and the medical treatment on quality-of-life parameters should be performed. A sub-analysis of our data that compared DAOH in HTx patients and BTT-LVAD patients waiting for more than one year and not being delisted in that time showed DAOH in the group without LVAD to be higher. This was expected, as the LVAD group undergoes an operation with a postoperative intensive care unit stay.

Our data suggest that BTT-LVAD implantation might not be favorable over sole HTx listing in hemodynamically stable heart failure patients as the DAOH and survival do not differ from HTx waiting. Additional information on improved quality of life, hospitalizations, and adverse events should be investigated in this patient cohort and might also contribute to the decision-making process in practical use. Another aspect besides the patient-centered outcomes concerns healthcare system effects due to hospitalizations and operations. Fewer days in hospital in terms of a lower DAOH means less financial burden. As we could see in this present study, the number of hospitalizations did not differ between the groups. In the country of this study site, Germany, there is no prioritization for HTx in BTT-LVAD patients as there is in the United States. Therefore, the waiting period is longer, as our data revealed in accordance with previous literature [20]. Almost 30% of our included patients did not have any further hospitalization in the year after LVAD implantation. Moreover, almost 30% of our patients received a HTx in the first year; two of them had an HU status due to LVAD-associated complications. It would be beneficial to find predictors for patients that will have a long-time benefit from the LVAD implantation and for those who will need a HTx shortly after implantation, therefore having two high-risk surgeries.

### Study Limitations

This study has several limitations. First, as our study is retrospective, the usual limitation for this study design appears. However, our database was constantly and prospectively updated. We reviewed all HTx and LVAD patients in our database and included HTx patients with a regular waiting list status. If a patient was transferred to HU status later in the process, they were also included for the LVAD patients. However, patients that died while waiting for HTx were not included, as we used the database containing all HTx patients that received a HTx. This aspect is a crucial limitation and must also be regarded for our survival analysis. In the LVAD database, we included all INTERMACS ≥4 patients and excluded patients with a DT intention at the time of implantation. Additionally, patients that were listed for transplantation more than one month before LVAD implantation were excluded, as we aimed to exclude LVAD implantation as a second-choice option. For better comparability, we performed propensity score matching. Through this process, the second limitation is the modest sample size. As this study was performed in a single center, following studies with greater sample sizes are necessary. Thirdly, for the assessment of our parameter DAOH, we cannot exclude external hospitalizations of the included patients. However, all LVAD and HTx-waiting patients were very closely connected to our center, so it is unlikely that these patients were hospitalized elsewhere. Lastly, we reviewed only a one-year follow-up after the decision for regular HTx waiting list or after LVAD implantation. Studies with long-time follow ups are crucial for generalizability and translation of the results into clinical practice.

## 5. Conclusions

In the present study, we showed that both decisions, to wait for a HTx or to undergo a LVAD implantation as a BTT (maximum one month after HTx listing), do not differ in terms of DAOH and survival in the first year after the decision. These data suggest that early LVAD implantation in patients eligible for HTx and still being hemodynamically stable might have no advantage over sole HTx listing. Investigations on quality of life after both decisions should follow. We cannot draw conclusions to enhance patient selection for each decision with this present study, especially with regard to the very low number of included patients and the long study duration of ten years with different devices and probably different pharmacologic treatments. It is crucial to verify and reproduce the results of this study in larger cohorts with a longer follow-up period or even with a prospective (randomized) design to gather further evidence in this important field.

## Figures and Tables

**Table 1:** Patient characteristics before and after PSM. PSM = propensity score matching; LVAD = left ventricular assist device; HTx = heart transplantation; BMI = body mass index; ICM = ischemic cardiomyopathy; DCM = dilatative cardiomyopathy; NYHA = New York Heart Association; LVEF = left ventricular ejection fraction.

	Before PSM			After PSM		
	LVAD Group (*n* = 21)	HTx Group (*n* = 44)	*p*-Value	LVAD Group (*n* = 17)	HTx Group (*n* = 17)	*p*-Value
Age, mean (SD)	59.97 (6.2)	55.69 (11.2)	0.108	59.92 (6.7)	57.70 (7.0)	0.352
Male sex, *n* (%)	17 (80.9)	26 (59.1))	0.08	13 (76.5)	12 (70.6)	0.697
BMI, mean (SD)	28.75 (4.9)	25.96 (4.2)	0.021	27.30 (3.8)	27.49 (3.4)	0.882
Renal failure, *n* (%)	6 (28.6)	19 (43.2)	0.258	5 (29.4)	7 (41.2)	0.473
diagnosis						
ICM, *n* (%)	16 (76.2)	17 (38.6)	0.07	12 (70.6)	8 (47.1)	0.163
DCM, *n* (%)	5 (23.8)	22 (50.0)		5 (29.4)	9 (52.9)	
others, *n* (%)	0 (0)	5 (11.4)		0 (0)	0 (0)	
NYHA						
NYHA I, *n* (%)	1 (4.8)	1 (2.3)	0.608	0 (0)	1 (5.9)	0.415
NYHA II, *n* (%)	0 (0)	0 (0)		0 (0)	0 (0)	
NYHA III, *n* (%)	14 (66.7)	24 (54.5)		11 (64.7)	8 (47.1)	
NYHA IV, *n* (%)	6 (28.5)	19 (43.2)		6 (35.3)	8 (47.1)	
LVEF						
Preserved (>50%), *n* (%)	0 (0)	1 (2.3)	0.472	0 (0)	0 (0)	
Mild reduced (40–49%), *n* (%)	0 (0)	2 (4.5)		0 (0)	0 (0)	
Reduced (<40%), *n* (%)	21 (100)	41 (93.2)		17 (100)	17 (100)	

**Table 2:** Secondary endpoints. HTx = heart transplantation; LVAD = left ventricular assist device; HU = high-urgency status.

	LVAD Group (*n* = 17)	HTx Waiting (*n* = 17)
Survival		
1 y survival, *n* (%)	14 (82.4)	13 (76.5)
3 y survival, *n* (%)	13 (76.5)	10 (58.8)
Duration until death in days, median (IQR)	401 (1122)	314 (279)
HTx waiting period		
Duration until HTx in days, median (IQR)	256 (275)	179 (308)
HU-listed, *n* (%)	4 (23.5)	2 (11.8)
Delisted, *n* (%)	5 (29.4)	0 (0)
Hospitalizations within one year		
Number of stays, mean (SD)	3 (1.97)	2.53 (2.12)
Duration in days, mean (SD)	32.24 (28.39)	25.77 (25.17)

## Data Availability

All relevant data for the understanding and interpretation of this study are included in the present manuscript.

## References

[1] McDonagh T.A., Metra M., Adamo M., Gardner R.S., Baumbach A., Böhm M., Burri H., Butler J., Čelutkienė J., Chioncel O. (2021). 2021 ESC Guidelines for the Diagnosis and Treatment of Acute and Chronic Heart FailureDeveloped by the Task Force for the Diagnosis and Treatment of Acute and Chronic Heart Failure of the European Society of Cardiology (ESC) With the Special Contribution of the Heart Failure Association (HFA) of the ESC. Eur. Heart J..

[2] Heidenreich P.A., Bozkurt B., Aguilar D., Allen L.A., Byun J.J., Colvin M.M., Deswal A., Drazner M.H., Dunlay S.M., Evers L.R. (2022). 2022 AHA/ACC/HFSA Guideline for the Management of Heart Failure: A Report of the American College of Cardiology/American Heart Association Joint Committee on Clinical Practice Guidelines. Circulation.

[3] Starling R.C., Estep J.D., Horstmanshof D.A., Milano C.A., Stehlik J., Shah K.B., Bruckner B.A., Lee S., Long J.W., Selzman C.H. (2017). Risk Assessment and Comparative Effectiveness of Left Ventricular Assist Device and Medical Management in Ambulatory Heart Failure Patients: The ROADMAP Study 2-Year Results. JACC Heart Fail.

[4] Rose E.A., Gelijns A.C., Moskowitz A.J., Heitjan D.F., Stevenson L.W., Dembitsky W., Long J.W., Ascheim D.D., Tierney A.R., Levitan R.G. (2009). Long-Term Use of a Left Ventricular Assist Device for End-Stage Heart Failure. N. Engl. J. Med..

[5] Theochari C.A., Michalopoulos G., Oikonomou E.K., Giannopoulos S., Doulamis I.P., Villela M.A., Kokkinidis D.G. (2018). Heart Transplantation versus Left Ventricular Assist Devices as Destination Therapy or Bridge to Transplantation for 1-Year Mortality: A Systematic Review and Meta-Analysis. Ann. Cardiothorac. Surg..

[6] Hullin R., Meyer P., Yerly P., Kirsch M. (2022). Cardiac Surgery in Advanced Heart Failure. J. Clin. Med..

[7] Guglin M., Zucker M.J., Borlaug B.A., Breen E., Cleveland J., Johnson M.R., Panjrath G.S., Patel J.K., Starling R.C., Bozkurt B. (2020). Evaluation for Heart Transplantation and LVAD Implantation: JACC Council Perspectives. J. Am. Coll. Cardiol..

[8] Stevenson L.W., Pagani F.D., Young J.B., Jessup M., Miller L., Kormos R.L., Naftel D.C., Ulisney K., Desvigne-Nickens P., Kirklin J.K. (2009). INTERMACS Profiles of Advanced Heart Failure: The Current Picture. J. Heart Lung. Transpl..

[9] Shah K.B., Starling R.C., Rogers J.G., Horstmanshof D.A., Long J.W., Kasirajan V., Stehlik J., Chuang J., Farrar D.J., Estep J.D. (2018). Left Ventricular Assist Devices versus Medical Management in Ambulatory Heart Failure Patients: An Analysis of INTERMACS Profiles 4 and 5 to 7 from the ROADMAP Study. J. Heart Lung. Transpl..

[10] Garbade J., Barten M.J., Bittner H.B., Mohr F.W. (2013). Heart Transplantation and Left Ventricular Assist Device Therapy: Two Comparable Options in End-Stage Heart Failure?. Clin. Cardiol..

[11] Miller L., Birks E., Guglin M., Lamba H., Frazier O.H. (2019). Use of Ventricular Assist Devices and Heart Transplantation for Advanced Heart Failure. Circ. Res..

[12] Mehra M.R., Naka Y., Uriel N., Goldstein D.J., Cleveland J.C., Colombo P.C., Walsh M.N., Milano C.A., Patel C.B., Jorde U.P. (2017). A Fully Magnetically Levitated Circulatory Pump for Advanced Heart Failure. N. Engl. J. Med..

[13] Mehra M.R., Goldstein D.J., Cleveland J.C., Cowger J.A., Hall S., Salerno C.T., Naka Y., Horstmanshof D., Chuang J., Wang A. (2022). Five-Year Outcomes in Patients With Fully Magnetically Levitated vs Axial-Flow Left Ventricular Assist Devices in the MOMENTUM 3 Randomized Trial. JAMA.

[14] Jerath A., Austin P.C., Wijeysundera D.N. (2019). Days Alive and Out of HospitalValidation of a Patient-Centered Outcome for Perioperative Medicine. Anesthesiology.

[15] Roth S., M’Pembele R., Stroda A., Voit J., Lurati Buse G., Sixt S.U., Westenfeld R., Polzin A., Rellecke P., Tudorache I. (2022). Days Alive and out of Hospital after Left Ventricular Assist Device Implantation. ESC Heart Fail..

[16] Zhang B., Guo S., Ning J., Li Y., Liu Z. (2021). Continuous-Flow Left Ventricular Assist Device versus Orthotopic Heart Transplantation in Adults with Heart Failure: A Systematic Review and Meta-Analysis. Ann. Cardiothorac. Surg..

[17] Hussain A., Kashem M.A., Suryapalam M., Kehara H., Toyoda Y. (2022). Comparison of LVAD as BTT with Direct Heart Transplantation. J. Heart Lung Transplant..

[18] Attisani M., Centofanti P., la Torre M., Boffini M., Ricci D., Ribezzo M., Baronetto A., Rinaldi M. (2012). Advanced Heart Failure in Critical Patients (INTERMACS 1 and 2 Levels): Ventricular Assist Devices or Emergency Transplantation?. Interact Cardiovasc. Thorac. Surg..

[19] M’Pembele R., Roth S., Stroda A., Buse G.L., Sixt S.U., Westenfeld R., Polzin A., Rellecke P., Tudorache I., Hollmann M.W. (2022). Life Impact of VA-ECMO Due to Primary Graft Dysfunction in Patients after Orthotopic Heart Transplantation. ESC Heart Fail..

[20] Immohr M.B., Boeken U., Mueller F., Prashovikj E., Morshuis M., Böttger C., Aubin H., Gummert J., Akhyari P., Lichtenberg A. (2021). Complications of Left Ventricular Assist Devices Causing High Urgency Status on Waiting List: Impact on Outcome after Heart Transplantation. ESC Heart Fail..

